# Breast Volume Measurements and Objectivations for Determination of Symmetry: A Retrospective Study in a Large Study Population

**DOI:** 10.1007/s00266-026-06058-w

**Published:** 2026-07-01

**Authors:** Renee C. Killaars, Daan G E. Janssen, A A. Piatkowski de Grzymala

**Affiliations:** 1 Department of Plastic, Reconstructive and Hand Surgery, Maastricht University Medical Centre+, P. Debyelaan 25, 6229 HX Maastricht, The Netherlands; 2 Viecuri Medical Centre, Venlo, The Netherlands

**Keywords:** Breast symmetry, 3D imaging, Volume measurement, VECTRA

## Abstract

**Background:**

Breast symmetry plays a key role in breast reconstructive surgery. In the healthy population, volume asymmetry is common. This study aimed to better understand how breast symmetry is perceived and to compare subjective estimation with objective measurements of breast symmetry in the healthy participants.

**Methods:**

A retrospective cross-sectional study included healthy participants who underwent VECTRA 3-dimentional breast imaging. Breast volume measurements were obtained using the VECTRA system. Five plastic surgeons and five laypersons assessed volume symmetry subjectively.

**Results:**

Of 209 healthy participants, 100 were randomly selected for assessment. Mean left and right breast volumes were 636.8 cc and 664.8 cc, respectively, with a mean relative volume difference of 16.0% (SD 12.7). No clear threshold of relative breast volume difference was found in which the majority of plastic surgeons or laypersons defined breast asymmetry. Even with large volume differences quantified by 3D VECTRA measurement, breasts were perceived as symmetrical by both plastic surgeons and laypersons. Inter-observer agreement was poor for both groups (Fleiss’ kappa: surgeons 0.23; laypersons 0.30). Agreement with 3D measurements regarding the larger breast was low (surgeons: accuracy 53.8%, Cohen’s kappa 0.08; laypersons: accuracy 48.6%, Cohen’s kappa –0.07).

**Conclusions:**

Subjective assessments of breast symmetry by both experts and laypersons poorly correlate with objective 3D imaging volume measurements. Symmetry perception likely extends beyond volume alone, influenced by factors such as breast shape, projection, and nipple position. While 3D imaging provides reliable volume measurements and preoperative consultations, it alone may not enhance patient satisfaction regarding perceived symmetry.

**Level of Evidence IV:**

This journal requires that authors assign a level of evidence to each article. For a full description of these Evidence-Based Medicine ratings, please refer to the Table of Contents or the online Instructions to Authors  www.springer.com/00266.

## Introduction

Breast symmetry is a common concern for patients undergoing breast surgery as well as for plastic surgeons. Few studies have described objective findings regarding breast symmetry. Losken et al. found that, on average, the left breast is larger than the right, with differences in size and shape being consistent but somewhat unpredictable [1]. In general, breast symmetry can be influenced by factors such as age, BMI, hormonal changes, and other individual characteristics [2, 3]. Breast symmetry is evaluated during the pre-operative, intra-operative, and postoperative stages and is considered an important outcome. Despite the fact that asymmetry occurs in almost all women, achieving breast symmetry plays a key role in breast reconstructive surgery, as it has been shown to improve a patient’s body image and satisfaction with surgical outcomes [4].

During physical examination, both breast volume and symmetry are assessed. To improve accuracy, several objective techniques have been developed, including water displacement, casting, Grossman–Roudner measuring device, mammography, MRI imaging, CT imaging, or by three-dimensional (3D) imaging [5–9]. Systematic reviews have shown that the most objective assessment of the breast considers volume, shape, and surface, with 3D modeling and MRI being the most reliable tools [10].

Three-dimensional imaging is a non-invasive and valid method for determining breast measurements. It can accurately calculate breast volume using multiple anatomical landmarks placed on a 3D image of the breast [11]. Previous studies have reported the use of 3D imaging to assess breast (a)symmetry after reconstruction, in patients with idiopathic scoliosis and as an intra-operative assessment tool [12–14]. Bai et al. investigated whether VECTRA® XT 3D-SI could assess symmetry and analyze the correlation between volume and symmetry [15]. They found no strong statistical correlation between breast symmetry and volume differences. Intra-observer reproducibility was substantial to excellent for several 3D-SI measurements, independent of imaging posture, whereas inter-observer reproducibility was lower, indicating that 3D-SI in its present form is limited for assessing symmetry.

This study aims to investigate the role of 3D imaging in assessing breast symmetry, by investigating whether the subjective evaluations of breast symmetry by plastic surgeons and laypersons could be objectified by 3D imaging measurements. We also aim to gain better insight into the interpretation of breast volume symmetry between plastic surgeons and laypersons, ultimately improving patient expectations and informing discussions about surgical outcomes.

## Methods

### Patients, Setting, and Study Design

Patients who presented for a breast consultation at in the Netherlands between January 2015 and January 2019 were retrospectively analyzed. Patients were included in the study when they were 18 year or older and had a VECTRA three-dimensional photograph of their breast available. Patients who had a history of breast or thoracic surgery, as well as recent breast biopsies, were excluded. Patient files were checked to see whether they made objection against use of personal data for research. A total of 246 patients were available in the database, of whom 209 met the eligibility criteria. From these 209 patients, 100 were randomly selected for assessment. This study followed an retrospective cross-sectional comparative design and was performed in accordance with the Declaration of Helsinki and followed the STROBE guidelines [16].

### Three-Dimensional Imaging

The VECTRA XT 3D imaging system (Canfield Imaging Systems, Fairfield, N.J.) was used for making the 3D images of the breasts. The 3D images are made as a form of standard patient care at the outpatient clinic of plastic surgery department of. Patients were photographed in an upright standing position with arms akimbo, while the camera was maintained at chest height. The captured data set was saved and the VECTRA software automatically processed the images into a high-resolution three-dimensional model.

### Breast Volume and Breast Volume Symmetry Calculation

**Fig. 1:**
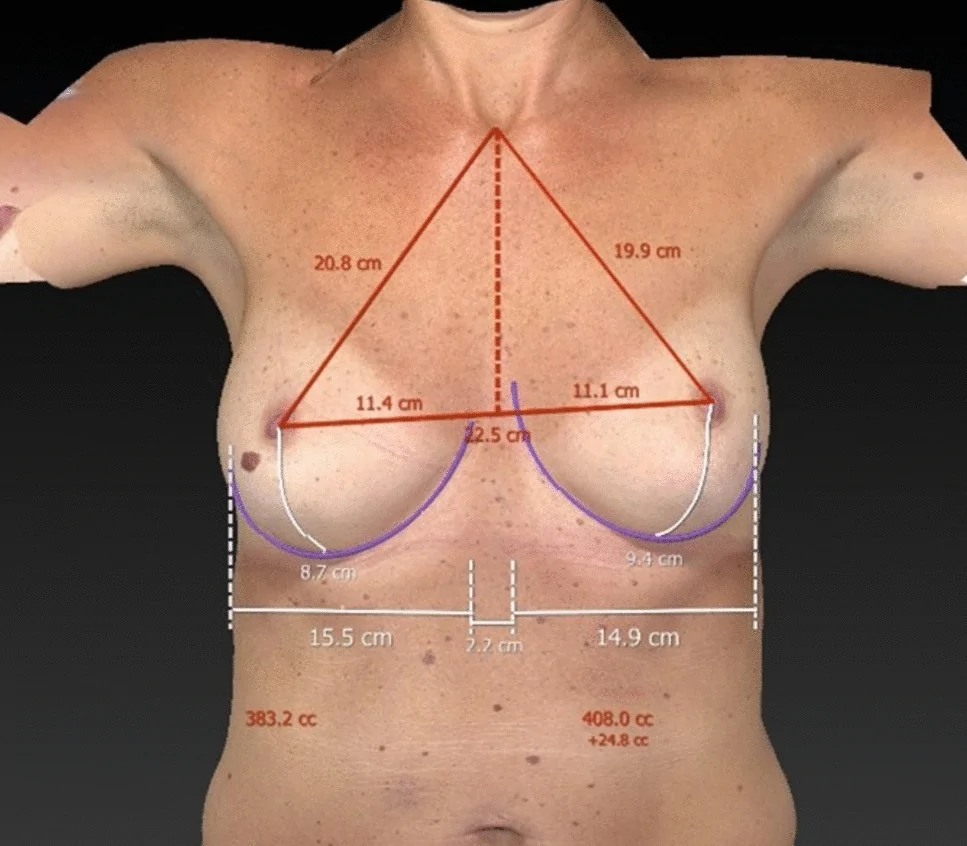
Three-dimensional imaging of the breast. Automatic calculation of breast volume with landmarks, using VECTRA Breast Sculptor

The processed 3D image was used for measurements of the breast. The researcher manually placed the following anatomic points in the image: jugulum (J), mid-clavicular (Cl), nipple (N), areola (Ar), median, and lateral border of the inframammary fold (IMF). From these anatomic points, the VECTRA system automatically calculated the breast measurements among the breast volume (see Fig. 1). Breast (a)symmetry was assessed using the relative volume difference. This was calculated by first determining the absolute volume difference between both breasts and then dividing this difference by the volume of the left breast, resulting in the relative volume difference.

### Breast Volume Symmetry Observations

One-hundred images of the breasts of study participants were assessed by five plastic surgeons to determine breast volume symmetry. The answer was dichotomous: symmetrical or asymmetrical. In case of asymmetry, the plastic surgeon also had to determine which breast had a bigger volume (left/right). Likewise five laypersons determined the breast volume symmetry.

### Statistical Analyses

Descriptive statistics (minimum, maximum, mean, and standard deviation) were calculated for age, height, BMI, and breast measurements. Fleiss’ kappa was calculated to assess inter-observer variability between plastic surgeons and laypersons. Cohen’s kappa was used to assess agreement between subjective assessments (by plastic surgeons and laypersons) and objective 3D VECTRA measurements of the side with greater breast volume in asymmetric patients. The strength of agreement was interpreted as follows: poor (<0), slight (0.00–0.20), fair (0.21–0.40), moderate (0.41–0.60), substantial (0.61–0.80), and almost perfect (0.81–1.00) [17]. All analyses and visualizations were performed using IBM SPSS Statistics version 29 and Microsoft Excel.

## Results

A total of 246 patients were identified in the database, of whom 209 met the eligibility criteria. Among these, 100 patients were randomly selected to be assessed by both plastic surgeons and laypersons and were therefore eligible for comparative analysis. Two patients were excluded from the final analysis due to being statistical outliers. These cases were identified as symmetric by the majority of plastic surgeons but showed implausibly large volume differences of 53.4% (490.1 cc) and 80.5% (397.0 cc), respectively, suggesting measurement or data entry errors. Thus, the final study cohort consisted of 98 patients.

Table 1 presents the demographic characteristics of the included patients. The mean age was 45.2 years (SD 13.0), with a mean height of 166.8 cm (SD 6.6) and a mean BMI of 26.5 kg/m^2^ (SD 4.2).

**Table 1:** Demographic characteristics of included patients (N=98)

Characteristics	Mean (Min–Max)	SD
Age (years)	45.2 (21.0-72.1)	13.0
Height (cm)	166.8 (153.0-18.0)	6.6
BMI (kg/m2)	26.5 (18.7-44.1)	4.2

Table 2 provides a summary of the breast measurements obtained via 3D surface imaging. The average breast volume was 636.8 cc on the left and 664.8 cc on the right, with a mean absolute volume difference of 102.5 cc (SD 90.2) and a mean relative volume difference of 16.0% (SD 12.7).

**Table 2:** Breast measurements of eligible patients (N=98)

Measurement	Mean (Min–Max)	SD
Left nipple–jugulum distance (cm)	26.3 (19.5-34.9)	4.0
Right nipple–jugulum distance (cm)	26.2 (18.7-36.9)	4.0
Width of left breast (cm)	17.1 (12.9-21.3)	1.9
Width of right breast (cm)	17.3 (12.9-22.2)	2.0
Left IMF–nipple projection (cm)	8.0 (4.1-12.1)	1.5
Right IMF–nipple projection (cm)	8.1 (2.8-18.0)	2.1
Left volume (cc)	636.8 (135.3-1544.0)	337.3
Right volume (cc)	664.8 (141.6-2024.9)	359.1
Volume difference (cc)	102.5 (0-522.1)	90.2
Relative volume difference	0.2 (0-0.9)	0.1
Volume difference percentage (%)	16.0 (0-91.9)	12.7

Inter-observer variability was assessed by calculating Fleiss’ kappa for both plastic surgeons and laypersons. Both are showing poor agreement with plastic surgeons having a Fleiss’ kappa of 0.23 (95%-CI 0.20-0.26) and laypersons 0.30 (95%-CI 0.28-0.32).

The subsequent sections present the main analytical results, comparing the assessments of symmetry between plastic surgeons, laypersons, and objective 3D volume measurements. Table 3 presents the classification of patients as symmetric or asymmetric by the majority vote of plastic surgeons and laypersons, stratified by relative volume difference. Notably, even at low volume differences (<10%), a substantial proportion of patients were classified as asymmetric, and at high volume differences (30–<35%) 80% were classified symmetric, indicating subjective assessment variability. Compared to plastic surgeons, laypersons classified a higher proportion of patients as symmetric, particularly in the 10–30% volume difference categories.

**Table 3:** Number of patients classified as symmetric or asymmetric by majority vote of plastic surgeons and laypersons, per relative breast volume difference measured by VECTRA 3D camera (N=98)

Relative Volume Difference	Plastic Surgeons		Laypersons		Total (n)
	Symmetric n (%)	Asymmetric n (%)	Symmetric n (%)	Asymmetric n (%)	
< 5%	11 (61.1%)	7 (38.9%)	13 (72.2%)	5 (27.8%)	18
5 - < 10%	8 (38.1%)	13 (61.9%)	12 (57.1%)	9 (42.9%)	21
10 - < 15%	7 (46.7%)	8 (53.3%)	9 (60.0%)	6 (40.0%)	15
15 - < 20%	4 (40.0%)	6 (60.0%)	5 (50.0%)	5 (50.0%)	10
20 - < 25%	7 (58.3%)	5 (41.7%)	10 (76.9%)	3 (23.1%)	13*
25 - < 30%	4 (33.3%)	8 (66.7%)	9 (75.0%)	3 (25.0%)	12
30 - < 35%	4 (80.0%)	1 (20.0%)	4 (80.0%)	1 (20.0%)	5
>35%	0 (0.0%)	4 (100.0%)	1 (25.0%)	3 (75.0%)	4
Total	45 (46.4%)	52 (53.6%)	63 (64.3%)	35 (35.7%)	98*

^*^Within the 20 – <25% volume difference category, there was one patient who received only four votes from plastic surgeons, which resulted in a tie, resulting in 12 patients in this category and 97 in total for plastic surgeons.

Table 4 evaluates agreement between plastic surgeons and 3D imaging on the side with the larger breast volume among the 52 patients classified as asymmetric. Agreement was moderate, with an accuracy of 53.8% (95%-CI 40.5–66.7) and a Cohen’s kappa of 0.08, indicating slight agreement. Sensitivity and specificity were 48.1% and 60.0%, respectively.

**Table 4:** Agreement between VECTRA 3D camera and plastic surgeons’ majority vote on the side with larger volume in patients classified as asymmetric by plastic surgeons (N=52)

3D Camera/Majority Vote	Left Larger	Right Larger	Total
Left larger	13	10	23
Right larger	14	15	29
Total	27	25	52

Sensitivity	48.1% (95%-CI 30.7-66.0)
Specificity	60.0% (95%-CI 40.7-76.6)
Positive predictive value	56.5% (95%-CI 36.8-74.4)
Negative predictive value	51.7% (95%-CI 34.4-68.8)
Accuracy	53.8% (95%-CI 40.5-66.7)
Cohen’s Kappa	0.08 (95%-CI -0.19-0.35)

Agreement between laypersons and 3D imaging (Table 5) in identifying the side with larger volume was lower than that of plastic surgeons, with an accuracy of 48.6% (95%-CI 33.0–64.4) and a Cohen’s kappa of –0.07, suggesting no agreement beyond chance. Sensitivity and specificity were 35.7% and 57.1%, respectively.

**Table 5:** Agreement between VECTRA 3D camera and laypersons majority vote on the side with larger volume in patients classified as asymmetric by laypersons (N=35)

3D Camera/Majority vote	Left larger	Right larger	Total
Left larger	5	9	14
Right larger	9	12	21
Total	14	21	35

Sensitivity	35.7% (95%-CI 16.3-61.2)
Specificity	57.1% (95%-CI 36.5-75.5)
Positive predictive value	35.7% (95%-CI 16.3-61.2)
Negative predictive value	57.1% (95%-CI 36.5-75.5)
Accuracy	48.6% (95%-CI 33.0-64.4)
Cohen’s Kappa	-0.07 (95%-CI -0.42-0.26)

**Fig. 2:**
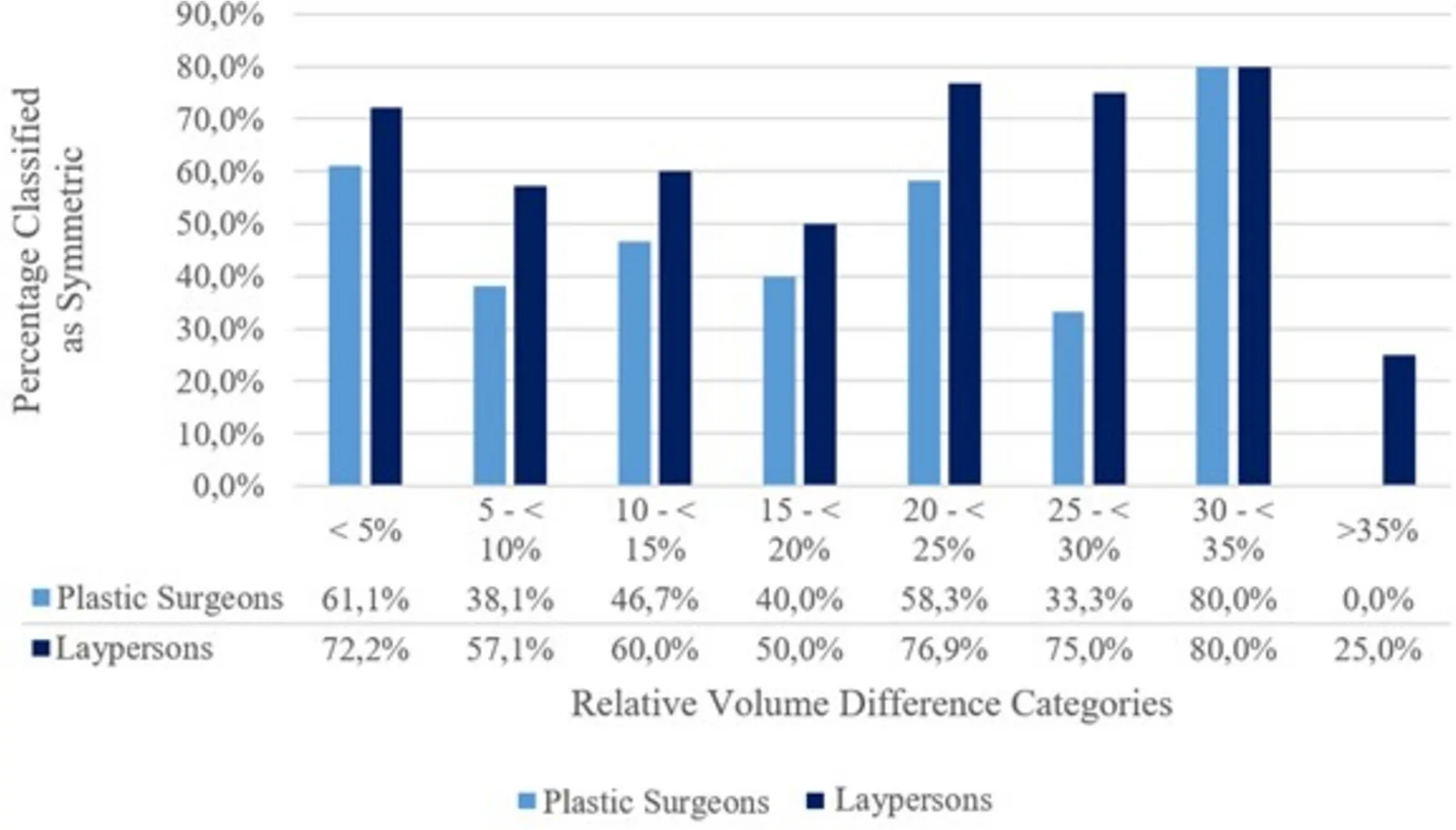
Percentage of patients classified as symmetric by majority vote of plastic surgeons or laypersons, per relative breast volume difference measured by VECTRA 3D camera. Graph illustrating the percentage of patients classified as having symmetrical breasts by majority vote of plastic surgeons or laypersons, stratified by categories of relative breast volume differences measured using VECTRA

Figure 2 combines the information of both Table 4 and Table 5, showing the percentage of patients classified as symmetric by either plastic surgeons’ or laypersons majority vote within each volume difference category, showcasing that in every category the laypersons have a higher symmetric rate than plastic surgeons, except for 30–< 35% volume difference where it is equal.

## Discussion

### Key Results

The aim of this study was to present objective measurements alongside subjective symmetry assessments by both plastic surgeons and laypersons, thereby analyzing the accuracy of visual evaluation. Our results show that laypersons classified more patients as symmetric compared to plastic surgeons (64.3% vs. 46.4%), particularly in the group with a relative volume difference of 10–<30%.

When examining agreement on identifying the larger breast within the asymmetric group, both plastic surgeons and laypersons demonstrated low accuracy (53.8% [95% CI 40.5–66.7] and 48.6% [95% CI 33.0–64.4], respectively) and low Cohen’s kappa values (0.08 [95% CI –0.19–0.35] and –0.07 [95% CI –0.42–0.26], respectively). This indicates that both groups had difficulty accurately assessing breast asymmetry. These findings are supported by the relatively low Fleiss’ kappa scores (0.23 [95% CI 0.20–0.26] for plastic surgeons and 0.30 [95% CI 0.28–0.32] for laypersons), further confirming low inter-observer agreement on symmetry.

### Interpretation

Previous studies have shown that when VECTRA 3D imaging is compared with other objective methods for volumetric assessment, such as MRI, it shows excellent reliability. Furthermore, when compared with subjective volume assessments, it shows no significant difference from estimations made by plastic surgeons [18]. However, considering the aim of this study, comparing VECTRA 3D imaging (objective) with subjective breast symmetry assessment, there appears to be a clear gap between objective volume measurements and visual (subjective) symmetry assessments. Even in cases with relatively large volume differences, many breasts were still perceived as symmetrical. Additionally, no clear threshold for volume difference was identified below which asymmetry typically goes unnoticed, by either laypersons or professionals, consistent with the findings of Bai et al [15].

Pham et al. compared a breast surface imaging system (VECTRA® XT 3D-SI) and breast contour measuring software (Mirror®) with the Harvard Cosmesis scale, a subjective surgeon assessment, and concluded that VECTRA® and Mirror® software have potential for objectifying breast symmetry in clinical practice [19]. However, in their current form, they are not yet ideal for routine clinical assessment of breast symmetry. The main limitations being the software assess symmetry as an average of the global surface symmetry, which can mask focal asymmetries of the breast. Additionally, they found that thoracic rotation affected the measurements and that symmetry measurements may be less accurate in large-breasted women, where ptosis or body mass index prevents the 3D camera from capturing the undersurface of the breasts [19].

Henseler et al. also compared postoperative subjective and objective assessment of breast symmetry using 3D imaging and found a highly significant relation between the two. Their 3D imaging software calculated symmetry scores based on differences between corresponding landmark points instead of global surface symmetry [20].

This suggests that visual perception of symmetry is influenced by more than just volume, as seen in our results. Additional factors such as nipple position, breast shape, skin laxity, or projection may play a significant role. Chan et al. described the influence of scoliosis on the perception of breast asymmetry, even when volume differences were small [21]. Moreover, Yip et al. reported no correlation between objectively measured postoperative volume symmetry and patient-reported satisfaction, further supporting the notion that perceived outcomes depend on more than volume alone [22]. Importantly, perceived symmetry also remains highly subjective, as reflected in the low inter-observer agreement among both plastic surgeons and laypersons.

These insights are clinically relevant. Although achieving volumetric symmetry is often a primary goal in reconstructive or aesthetic breast surgery, our findings demonstrate that volume assessment alone is insufficient. Yip et al. found that the preoperative information provided is the strongest predictor of patient satisfaction with reconstructed breasts [22]. Integrating objective tools such as 3D imaging into preoperative consultations could therefore help set more realistic expectations and reduce dissatisfaction related to perceived asymmetry after surgery.

### Limitations

This study has several limitations. While the VECTRA 3D system is proven reliable, it relies on manual placement of anatomical landmarks for volume calculation, introducing potential inter-user variability. Although the VECTRA imaging system has demonstrated reliability when compared to MRI [18], and the primary researcher was proficient with the software, the reliance on single observer precludes the assessment of inter-observer reliability and remains susceptible to manual error. O’Connell et al. evaluated inter-observer variation using the software and reported a mean relative volume difference of 0.11% between two observers, with differences of up to 5.78% across three repeated measurements [11]. Additionally, despite standardized positioning with arm akimbo, factors such as thoracic rotation and significant breast ptosis may further compromise measurement precision. Furthermore, subjective assessments of breast symmetry may have been influenced by individual observer biases or perceptual thresholds, particularly among laypersons who received no training.

The study was also limited to a single-center cohort, which may have introduced selection bias and limited generalizability. Finally, due to the cross-sectional design, it was not possible to evaluate the influence of time, surgical interventions, or hormonal fluctuations on breast volume symmetry.

## Conclusion

This study demonstrates a poor concordance between subjective assessments of breast symmetry, performed by both plastic surgeons and laypersons, and objective volume measurements obtained through three-dimensional (3D) imaging. Notably, even in cases with clear volumetric asymmetry, a substantial number of breasts were still perceived as symmetrical. Observers were unable to reliably identify which breast was volumetrically larger. These findings indicate that the perception of breast symmetry is influenced by multiple factors beyond volume alone, including nipple position, breast contour, projection, and individual perceptual thresholds.

The results highlight that, although 3D imaging provides valuable and highly reliable data for evaluating breast volume symmetry, its utility in assessing overall breast symmetry remains limited when used in isolation. However, these objective insights are of significant clinical value in preoperative planning and intra-operative decision-making. Objective measurements can guide the precise volume of symmetrizing reductions or the selection of implant sizes. Furthermore, providing patients with objective data can help align subjective perceptions with clinical reality, potentially refining the indications for surgery and preventing unnecessary secondary corrections in cases where perceived asymmetry is not reflected in volumetric data.

Future research should focus on refining objective symmetry assessment tools by integrating additional anatomical and aesthetic parameters. Correlating these objective measurements with patient-reported outcomes may help bridge the gap between clinical evaluation and patient satisfaction across diverse populations, including healthy individuals and those undergoing breast cancer treatment.
